# Supplementary material for: SMARTdb: An Integrated Database for Exploring Single-cell Multi-omics Data of Reproductive Medicine
Source: Genomics Proteomics Bioinformatics. 2024 Jan 10;22(3):qzae005. doi: 10.1093/gpbjnl/qzae005 (PMC12016030; doi:10.1093/gpbjnl/qzae005)
Supplement: qzae005_Supplementary_Data [file qzae005_supplementary_data.zip › Supplementary material captions.docx]

**Supplementary material**

**Figure S1 The Sankey plot shows the composition of datasets included in SMARTdb**

AZF, azoospermia factor; OA, obstructive azoospermia; NOA, Non-obstructive azoospermia.

**Figure S2 The DNA methylation levels and chromatin accessibility levels around the *RPL39L* promoter in fetal germ cells after 11 weeks**

The screenshots of Epigenome browser displaying the DNA methylation levels and chromatin accessibility levels around the *RPL39L* promoter in fetal germ cells after 11 weeks post fertilization. The gray rectangles highlight regions showing significant differences between germ cells and somatic niche cells.

**Table S1 Summary of curated datasets and covered time points**
