# Supplementary figures and images for: SMARTdb: An Integrated Database for Exploring Single-cell Multi-omics Data of Reproductive Medicine
Source: Genomics Proteomics Bioinformatics. 2024 Jan 10;22(3):qzae005. doi: 10.1093/gpbjnl/qzae005 (PMC12016030; doi:10.1093/gpbjnl/qzae005)

Figure S2

DNA methylation

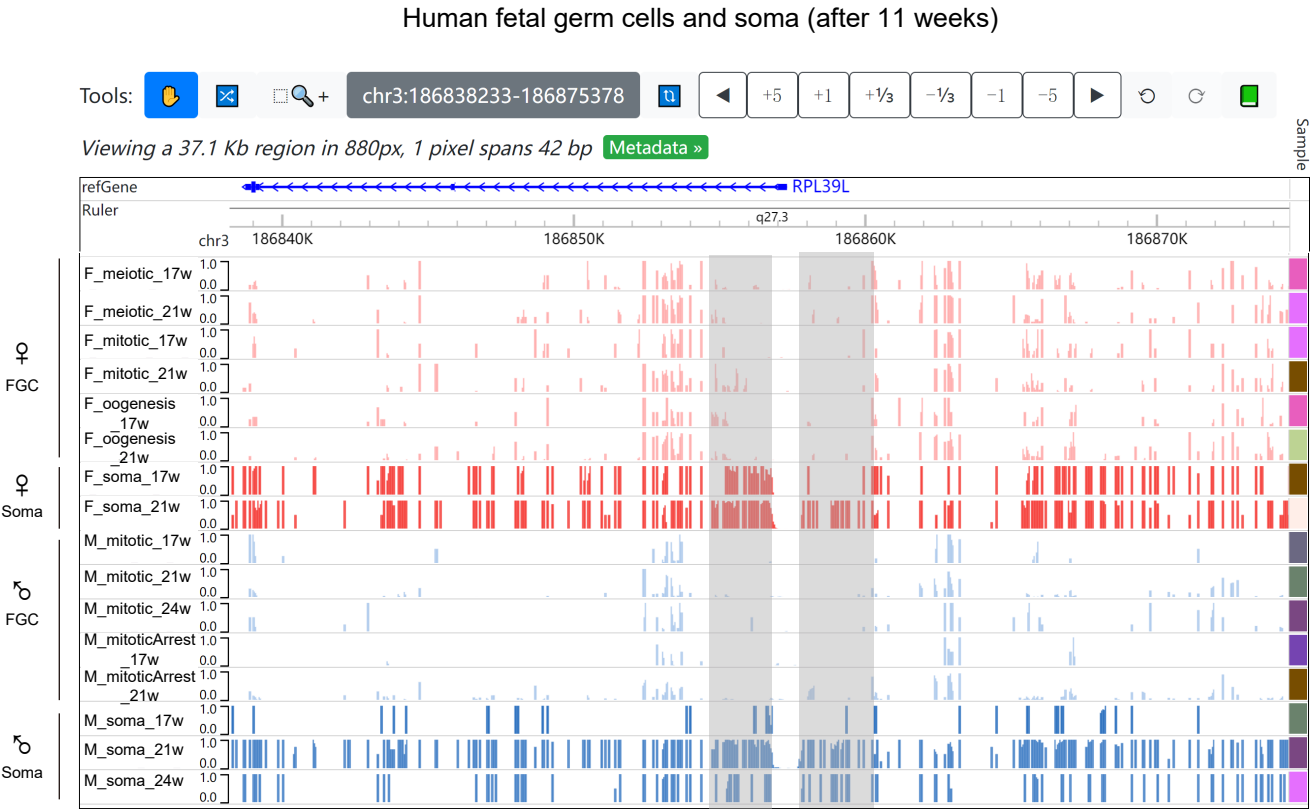

Chromatin accessibility

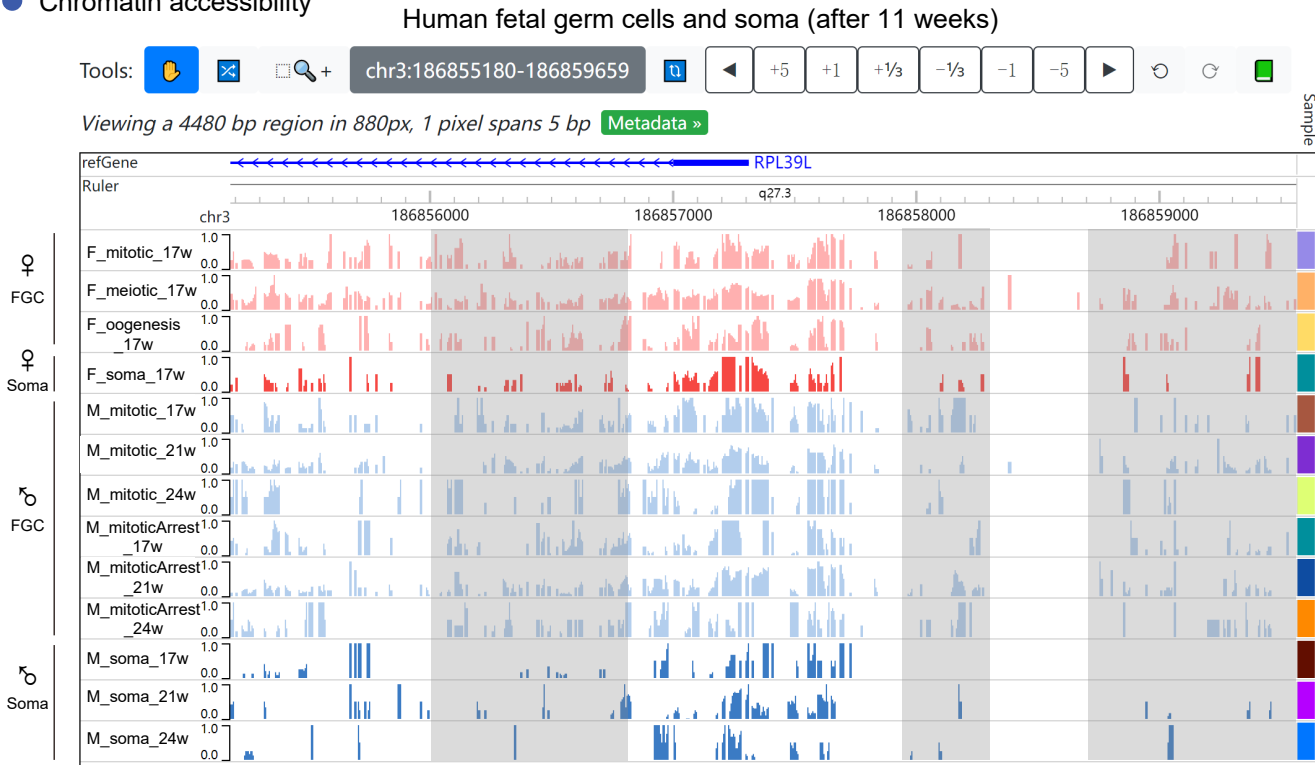

Supplement: qzae005_Supplementary_Data [file qzae005_supplementary_data.zip › Figure S2.pdf]

Figure S1

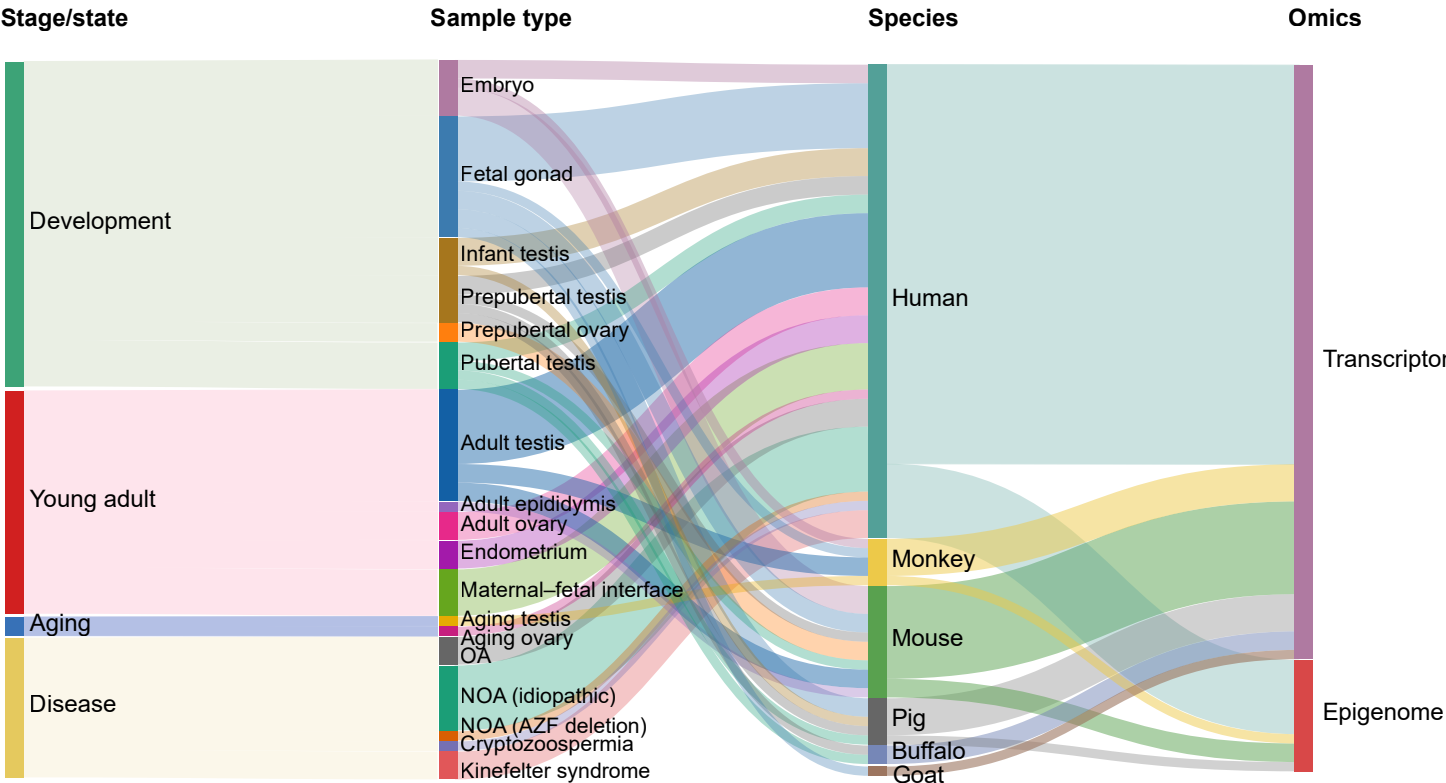

Supplement: qzae005_Supplementary_Data [file qzae005_supplementary_data.zip › Figure S1.pdf]
